# Tabes Messenterica—A Case

**Published:** 1885-07

**Authors:** Chas. Hicks

**Affiliations:** Dublin, Ga.


### TABES MESSENTERICA—A CASE.

REPORTED TO THE MEDICAL ASSOCIATION OF GEORGIA AT ITS THIRTY-SIXTH ANNUAL SESSION, SAVANNAH, APRIL, 1885, BY CHAS. HICKS, M. D., DUBLIN, GA.

Not for the purpose of adding lustre to the many bright pages of our annual "Transactions," but to show how easily a mistaken diagnosis may be clearly made out, is my apology for this paper.

G. T., age nine years, was brought by his mother into my office April 28th, 1884. His appearance was that of a child suffering from anæmia; but upon examination I found his abdomen very much distended and hard, with occasional spaces, which under palpation revealed liquid. Upon inquiring into the history of the case, I learned nothing important as to family history. The child had been unwell for twelve months, at times under the treatment of physicians, though not thought seriously ill; but more recently the mother's anxiety had been aroused on account of œdema of the lower extremities, and colicky pains which were distressing at night. When asked if the child had ever had sickness of a grave character, the mother replied, that at two years old he had a severe attack of pneumonia or of typhoid fever. During lactation the child nursed the bottle.

Cream of tartar and jallup were prescribed, to be given at intervals of two days, which, at the end of a week, had reduced the dropsy, leaving the abdomen filled with two tumors, which could be separated on a line from the junction of the median and the lower line bounding the epigastric region to the anterior superior spinous process of the left ilium. Supportive treatment was now instituted and bichloride of mercury and iodide of potassium given alternately, under whose influence the edges of these two tumors began to recede from each other. On the 15th of May the left tumor had withdrawn under the ribs, leaving a space of two and a half or three inches between the margin of the ribs and the edge of the large tumor. I was now able to follow distinctly the edge of the tumor all the way across the abdomen, and in the upper third of the right umbilical region I could grasp between my fingers a soft nodule, which I recognized as the distended gall bladder. The tumor stopped decreasing, appetite got to be ravenous, dropsy re-appeared, but was readily drained off with the first prescription. On the 20th of May my patient had begun to emaciate, up to which time he had stood the disease and his medicine pretty well. The tumor was now found to be immovable, apparently adhered to the walls of the abdomen. Seeing that I had an unusual case that was rapidly approaching a fatal termination, on the 22d of May I invited my neighboring fellow-practitioners "to see with me an unusually interesting case of liver trouble." The physician who first saw the patient with me thought that he had enlarged liver, with probably some other tumor, and recognized it as a similar trouble that had taken a son from his household—(the attending physicians used on the Doctor's child an aspirator without finding pus). Four days later—the day on which the patient died—another physician examined the tumor and thought that I had been dealing with a misplaced spleen. I thought all the while that I was treating enlarged liver and spleen, but the autopsy revealed the tubercular degeneration of the messenteric glands. The glands had enlarged to such an extent as to fill the abdomen, and had adhered to the peritoneum so firmly as to necessitate the use of a scalpel in separating the tumor from the walls of the abdomen. The small intestine from the duodenum to the colon, instead of retaining its identity as such, the coats of the bowel were in places obliterated and the winding track through the tumor resembled a rat hole in loose earth or dead wood. The strangest feature of the case to me is the ability of the bowels all the while to have exercised control over its contents. The tumor, in spots, had broken down and contained pus. My suspected gall bladder proved to be part of the tumor filled with pus. In the neighborhood of the site for Peyer's patches the mucous membrane was elevated and roughened, indicating an old cicatrix. The child at two years old probably had typhoid pneumonia, which, if not the artificial feeding, was the prime cause of the degenerated glands—maybe both. The liver was enlarged, but texture natural, spleen more than twice its natural size with evidences of having been very much enlarged, slight effusion in pericardium, lungs and kidneys normal.

Believing as I do, that an honest confession is not only good for the soul, but sometimes for suffering humanity as well, I have reported this case of mistaken diagnosis.

---

HEART BEATS.—Dr. W. B. Richardson, of London, says he was recently able to convey a considerable amount of conviction to an intelligent scholar by a simple experiment. The scholar was singing the praises of the "ruddy bumper," and saying he could not get through the day without it, when Dr. Richardson said to him: "Will you be good enough to feel my pulse as I stand here?" He did so. I said: "Count it carefully; what does it say?" "Your pulse says 74." I then sat down in a chair and asked him to count it again. He did so, and said: "Your pulse has gone down to 70." I then lay down on the lounge, and said: "Will you take it again?" He replied: "Why, it is only 64; what an extraordinary thing!" I then said: "When you lie down at night that is the way nature gives your heart rest. You know nothing about it, but that beating organ is resting to that extent; and if you reckon it up it is a great deal of rest, because in lying down the heart is doing ten strokes less a minute. Multiply that by 60 and it is 600; multiply it by eight hours, and within a fraction it is 5,000 strokes different; and as the heart is throwing six ounces of blood at every stroke, it makes a difference of 30,000 ounces of lifting during the night. When I lie down at night without any alcohol, that is the rest my heart gets. But when you take your wine or grog you do not allow that rest, for the influence of the alcohol is to increase the number of strokes, and instead of getting this rest, you put on something like 15,000 extra strokes, and the result is, you rise up very seedy and unfit for the next day's work till you have taken a little more of the 'ruddy bumper,' which you say is the soul of man below."—*Ex.*

